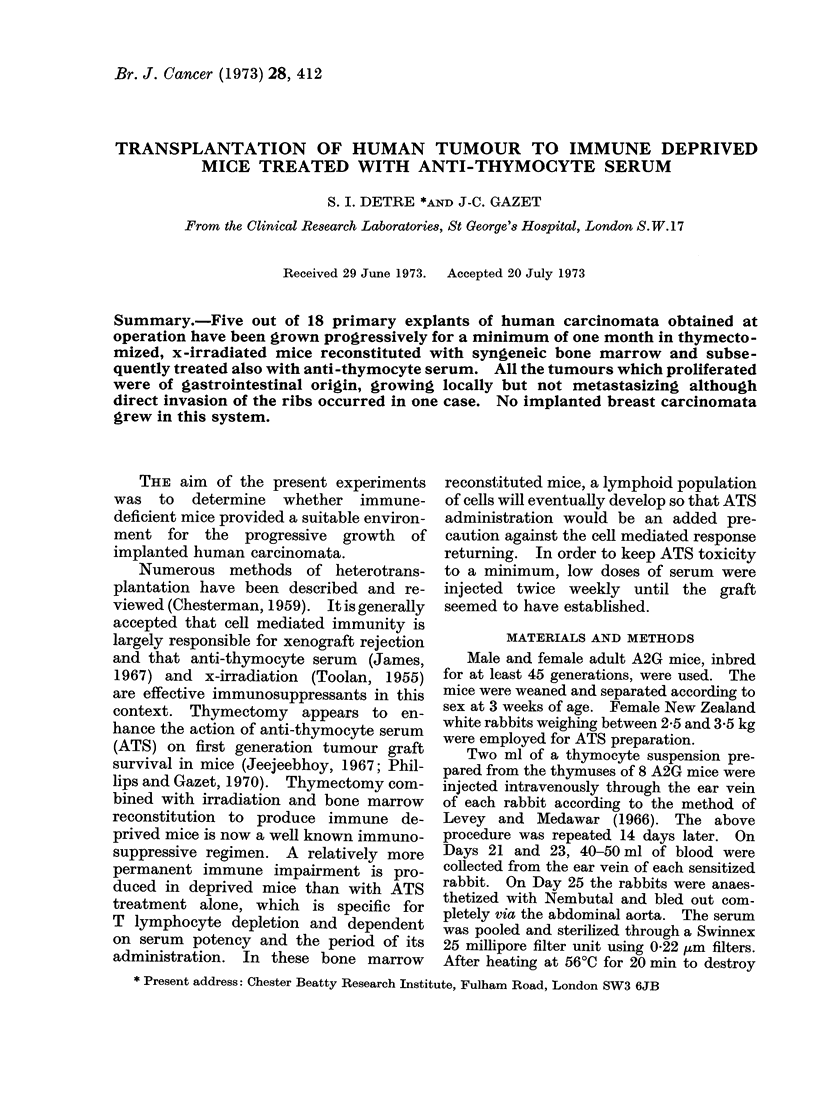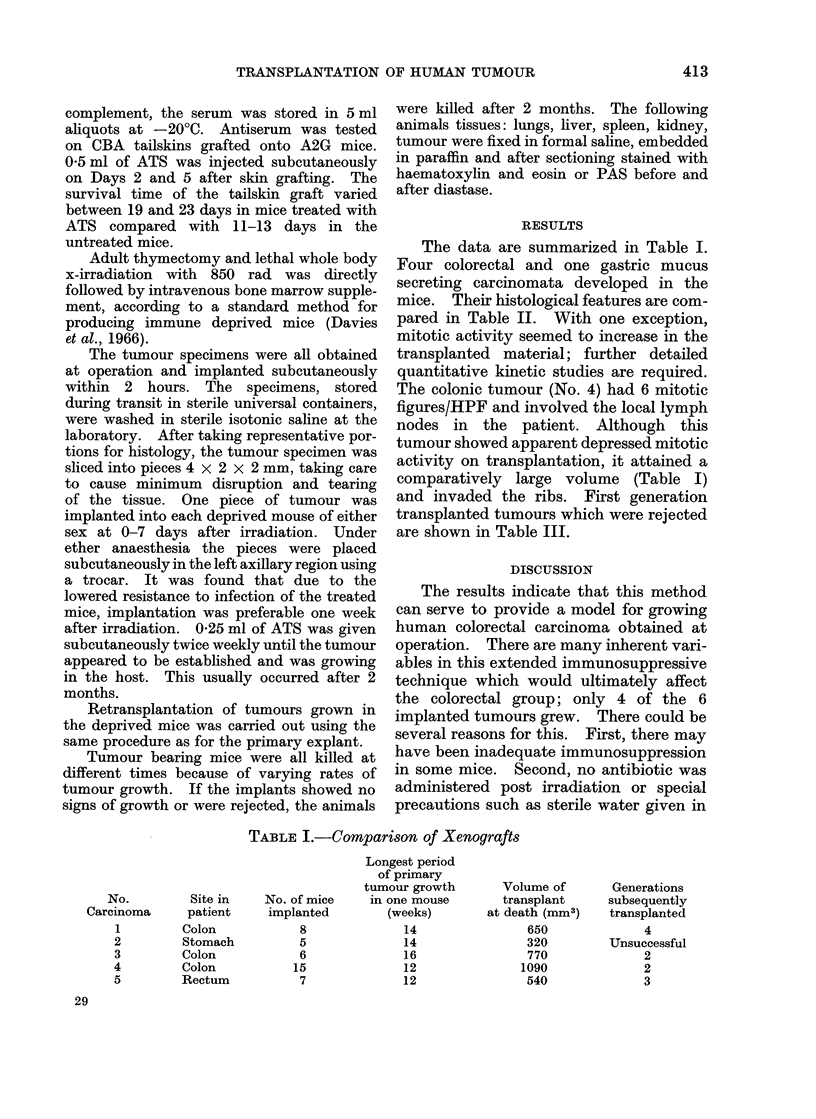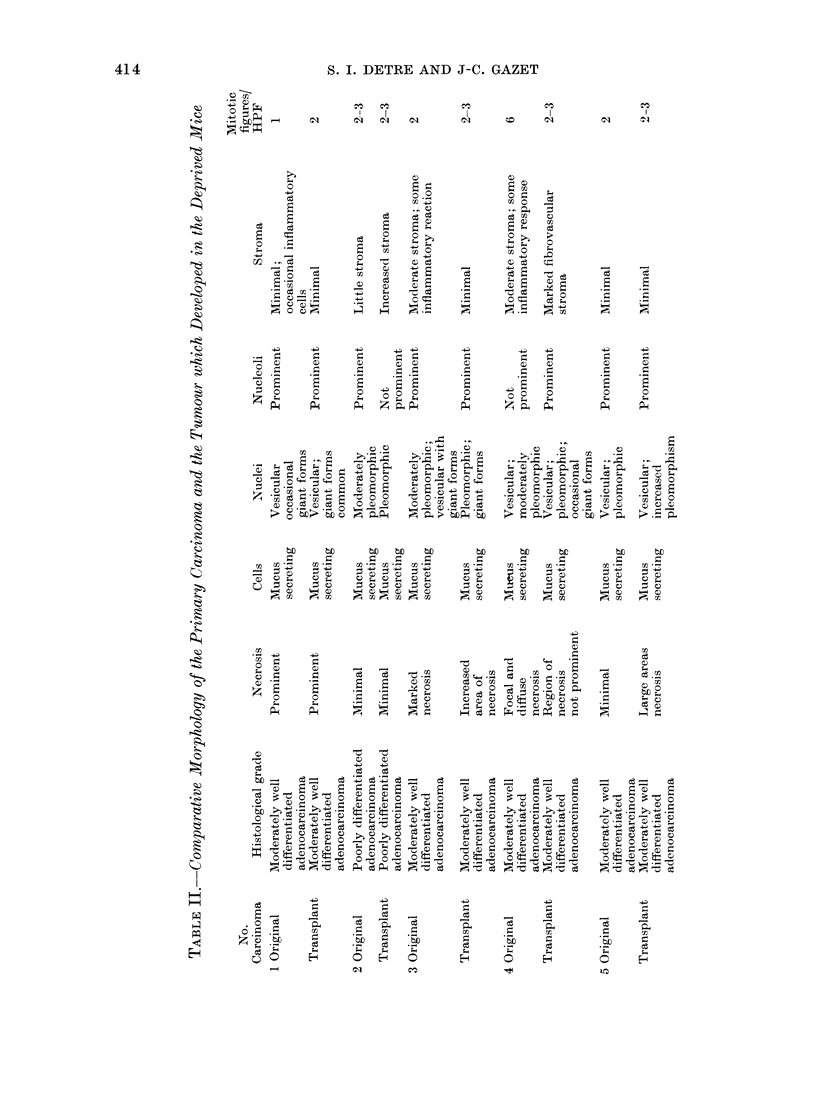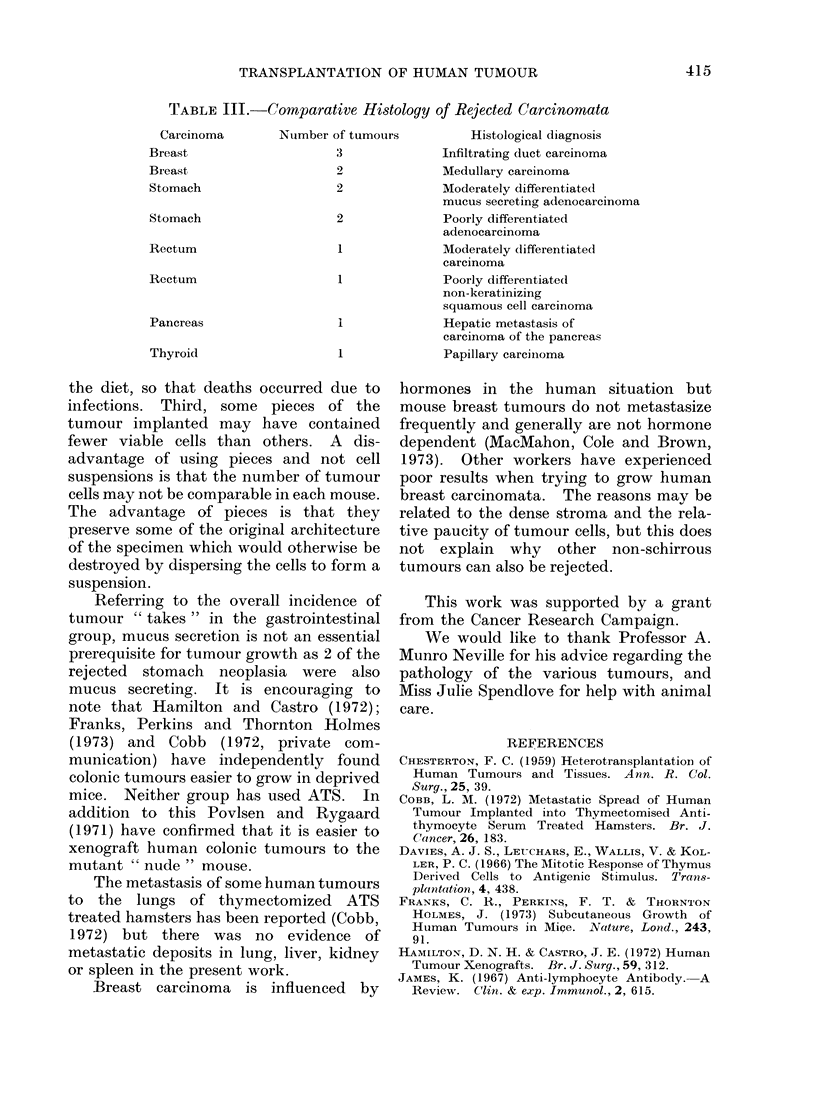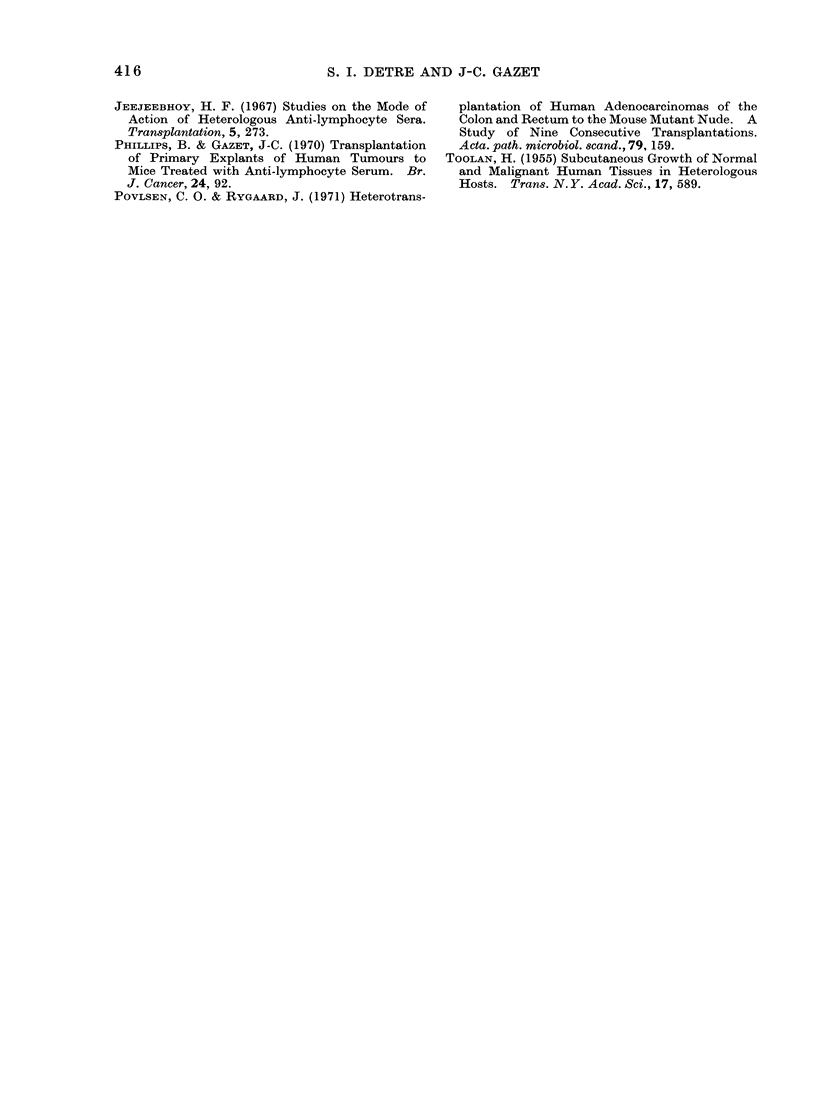# Transplantation of Human Tumour to Immune Deprived Mice Treated with Anti-thymocyte Serum

**DOI:** 10.1038/bjc.1973.166

**Published:** 1973-11

**Authors:** S. I. Detre, J-C. Gazet

## Abstract

Five out of 18 primary explants of human carcinomata obtained at operation have been grown progressively for a minimum of one month in thymectomized, x-irradiated mice reconstituted with syngeneic bone marrow and subsequently treated also with anti-thymocyte serum. All the tumours which proliferated were of gastrointestinal origin, growing locally but not metastasizing although direct invasion of the ribs occurred in one case. No implanted breast carcinomata grew in this system.


*Br. J. Cancer* (1973) **28**, 412

### TRANSPLANTATION OF HUMAN TUMOUR TO IMMUNE DEPRIVED MICE TREATED WITH ANTI-THYMOCYTE SERUM

S. I. DETRE *AND J-C. GAZET

*From the Clinical Research Laboratories, St George's Hospital, London S.W.17*

Received 29 June 1973. Accepted 20 July 1973

**Summary.—Five out of 18 primary explants of human carcinomata obtained at operation have been grown progressively for a minimum of one month in thymectomized, x-irradiated mice reconstituted with syngeneic bone marrow and subsequently treated also with anti-thymocyte serum. All the tumours which proliferated were of gastrointestinal origin, growing locally but not metastasizing although direct invasion of the ribs occurred in one case. No implanted breast carcinomata grew in this system.**

THE aim of the present experiments was to determine whether immune-deficient mice provided a suitable environment for the progressive growth of implanted human carcinomata.

Numerous methods of heterotransplantation have been described and reviewed (Chesterman, 1959). It is generally accepted that cell mediated immunity is largely responsible for xenograft rejection and that anti-thymocyte serum (James, 1967) and x-irradiation (Toolan, 1955) are effective immunosuppressants in this context. Thymectomy appears to enhance the action of anti-thymocyte serum (ATS) on first generation tumour graft survival in mice (Jeejeebhoy, 1967; Phillips and Gazet, 1970). Thymectomy combined with irradiation and bone marrow reconstitution to produce immune deprived mice is now a well known immunosuppressive regimen. A relatively more permanent immune impairment is produced in deprived mice than with ATS treatment alone, which is specific for T lymphocyte depletion and dependent on serum potency and the period of its administration. In these bone marrow reconstituted mice, a lymphoid population of cells will eventually develop so that ATS administration would be an added precaution against the cell mediated response returning. In order to keep ATS toxicity to a minimum, low doses of serum were injected twice weekly until the graft seemed to have established.

### MATERIALS AND METHODS

Male and female adult A2G mice, inbred for at least 45 generations, were used. The mice were weaned and separated according to sex at 3 weeks of age. Female New Zealand white rabbits weighing between 2·5 and 3·5 kg were employed for ATS preparation.

Two ml of a thymocyte suspension prepared from the thymuses of 8 A2G mice were injected intravenously through the ear vein of each rabbit according to the method of Levey and Medawar (1966). The above procedure was repeated 14 days later. On Days 21 and 23, 40–50 ml of blood were collected from the ear vein of each sensitized rabbit. On Day 25 the rabbits were anaesthetized with Nembutal and bled out completely *via* the abdominal aorta. The serum was pooled and sterilized through a Swinnex 25 millipore filter unit using 0·22 $\mu$m filters. After heating at 56°C for 20 min to destroy

* Present address: Chester Beatty Research Institute, Fulham Road, London SW3 6JB

complement, the serum was stored in 5 ml aliquots at −20°C. Antiserum was tested on CBA tailskins grafted onto A2G mice. 0·5 ml of ATS was injected subcutaneously on Days 2 and 5 after skin grafting. The survival time of the tailskin graft varied between 19 and 23 days in mice treated with ATS compared with 11–13 days in the untreated mice.

Adult thymectomy and lethal whole body x-irradiation with 850 rad was directly followed by intravenous bone marrow supplement, according to a standard method for producing immune deprived mice (Davies *et al.*, 1966).

The tumour specimens were all obtained at operation and implanted subcutaneously within 2 hours. The specimens, stored during transit in sterile universal containers, were washed in sterile isotonic saline at the laboratory. After taking representative portions for histology, the tumour specimen was sliced into pieces 4 × 2 × 2 mm, taking care to cause minimum disruption and tearing of the tissue. One piece of tumour was implanted into each deprived mouse of either sex at 0–7 days after irradiation. Under ether anaesthesia the pieces were placed subcutaneously in the left axillary region using a trocar. It was found that due to the lowered resistance to infection of the treated mice, implantation was preferable one week after irradiation. 0·25 ml of ATS was given subcutaneously twice weekly until the tumour appeared to be established and was growing in the host. This usually occurred after 2 months.

Retransplantation of tumours grown in the deprived mice was carried out using the same procedure as for the primary explant.

Tumour bearing mice were all killed at different times because of varying rates of tumour growth. If the implants showed no signs of growth or were rejected, the animals were killed after 2 months. The following animals tissues: lungs, liver, spleen, kidney, tumour were fixed in formal saline, embedded in paraffin and after sectioning stained with haematoxylin and eosin or PAS before and after diastase.

### RESULTS

The data are summarized in Table I. Four colorectal and one gastric mucus secreting carcinomata developed in the mice. Their histological features are compared in Table II. With one exception, mitotic activity seemed to increase in the transplanted material; further detailed quantitative kinetic studies are required. The colonic tumour (No. 4) had 6 mitotic figures/HPF and involved the local lymph nodes in the patient. Although this tumour showed apparent depressed mitotic activity on transplantation, it attained a comparatively large volume (Table I) and invaded the ribs. First generation transplanted tumours which were rejected are shown in Table III.

### DISCUSSION

The results indicate that this method can serve to provide a model for growing human colorectal carcinoma obtained at operation. There are many inherent variables in this extended immunosuppressive technique which would ultimately affect the colorectal group; only 4 of the 6 implanted tumours grew. There could be several reasons for this. First, there may have been inadequate immunosuppression in some mice. Second, no antibiotic was administered post irradiation or special precautions such as sterile water given in

TABLE I.—*Comparison of Xenografts*

| No. Carcinoma | Site in patient | No. of mice implanted | Longest period of primary tumour growth in one mouse (weeks) | Volume of transplant at death (mm³) | Generations subsequently transplanted |
|---|---|---|---|---|---|
| 1 | Colon | 8 | 14 | 650 | 4 |
| 2 | Stomach | 5 | 14 | 320 | Unsuccessful |
| 3 | Colon | 6 | 16 | 770 | 2 |
| 4 | Colon | 15 | 12 | 1090 | 2 |
| 5 | Rectum | 7 | 12 | 540 | 3 |

TABLE II.—*Comparative Morphology of the Primary Carcinoma and the Tumour which Developed in the Deprived Mice*

| No. Carcinoma | Histological grade | Necrosis | Cells | Nuclei | Nucleoli | Stroma | Mitotic figures/ HPF |
|---|---|---|---|---|---|---|---|
| 1 Original | Moderately well differentiated adenocarcinoma | Prominent | Mucus secreting | Vesicular occasional giant forms | Prominent | Minimal; occasional inflammatory cells | 1 |
| Transplant | Moderately well differentiated adenocarcinoma | Prominent | Mucus secreting | Vesicular; giant forms common | Prominent | Minimal | 2 |
| 2 Original | Poorly differentiated adenocarcinoma | Minimal | Mucus secreting | Moderately pleomorphic | Prominent | Little stroma | 2–3 |
| Transplant | Poorly differentiated adenocarcinoma | Minimal | Mucus secreting | Pleomorphic | Not prominent | Increased stroma | 2–3 |
| 3 Original | Moderately well differentiated adenocarcinoma | Marked necrosis | Mucus secreting | Moderately pleomorphic; vesicular with giant forms | Prominent | Moderate stroma; some inflammatory reaction | 2 |
| Transplant | Moderately well differentiated adenocarcinoma | Increased area of necrosis | Mucus secreting | Pleomorphic; giant forms | Prominent | Minimal | 2–3 |
| 4 Original | Moderately well differentiated adenocarcinoma | Focal and diffuse necrosis | Mucus secreting | Vesicular; moderately pleomorphic | Not prominent | Moderate stroma; some inflammatory response | 6 |
| Transplant | Moderately well differentiated adenocarcinoma | Region of necrosis not prominent | Mucus secreting | Vesicular; pleomorphic; occasional giant forms | Prominent | Marked fibrovascular stroma | 2–3 |
| 5 Original | Moderately well differentiated adenocarcinoma | Minimal | Mucus secreting | Vesicular; pleomorphic | Prominent | Minimal | 2 |
| Transplant | Moderately well differentiated adenocarcinoma | Large areas necrosis | Mucus secreting | Vesicular; increased pleomorphism | Prominent | Minimal | 2–3 |

TABLE III.—*Comparative Histology of Rejected Carcinomata*

| Carcinoma | Number of tumours | Histological diagnosis |
|---|---|---|
| Breast | 3 | Infiltrating duct carcinoma |
| Breast | 2 | Medullary carcinoma |
| Stomach | 2 | Moderately differentiated mucus secreting adenocarcinoma |
| Stomach | 2 | Poorly differentiated adenocarcinoma |
| Rectum | 1 | Moderately differentiated carcinoma |
| Rectum | 1 | Poorly differentiated non-keratinizing squamous cell carcinoma |
| Pancreas | 1 | Hepatic metastasis of carcinoma of the pancreas |
| Thyroid | 1 | Papillary carcinoma |

the diet, so that deaths occurred due to infections. Third, some pieces of the tumour implanted may have contained fewer viable cells than others. A disadvantage of using pieces and not cell suspensions is that the number of tumour cells may not be comparable in each mouse. The advantage of pieces is that they preserve some of the original architecture of the specimen which would otherwise be destroyed by dispersing the cells to form a suspension.

Referring to the overall incidence of tumour " takes " in the gastrointestinal group, mucus secretion is not an essential prerequisite for tumour growth as 2 of the rejected stomach neoplasia were also mucus secreting. It is encouraging to note that Hamilton and Castro (1972); Franks, Perkins and Thornton Holmes (1973) and Cobb (1972, private communication) have independently found colonic tumours easier to grow in deprived mice. Neither group has used ATS. In addition to this Povlsen and Rygaard (1971) have confirmed that it is easier to xenograft human colonic tumours to the mutant " nude " mouse.

The metastasis of some human tumours to the lungs of thymectomized ATS treated hamsters has been reported (Cobb, 1972) but there was no evidence of metastatic deposits in lung, liver, kidney or spleen in the present work.

Breast carcinoma is influenced by hormones in the human situation but mouse breast tumours do not metastasize frequently and generally are not hormone dependent (MacMahon, Cole and Brown, 1973). Other workers have experienced poor results when trying to grow human breast carcinomata. The reasons may be related to the dense stroma and the relative paucity of tumour cells, but this does not explain why other non-schirrous tumours can also be rejected.

This work was supported by a grant from the Cancer Research Campaign.

We would like to thank Professor A. Munro Neville for his advice regarding the pathology of the various tumours, and Miss Julie Spendlove for help with animal care.

### REFERENCES

CHESTERTON, F. C. (1959) Heterotransplantation of Human Tumours and Tissues. *Ann. R. Col. Surg.*, **25**, 39.

COBB, L. M. (1972) Metastatic Spread of Human Tumour Implanted into Thymectomised Antithymocyte Serum Treated Hamsters. *Br. J. Cancer*, **26**, 183.

DAVIES, A. J. S., LEUCHARS, E., WALLIS, V. & KOLLER, P. C. (1966) The Mitotic Response of Thymus Derived Cells to Antigenic Stimulus. *Transplantation*, **4**, 438.

FRANKS, C. R., PERKINS, F. T. & THORNTON HOLMES, J. (1973) Subcutaneous Growth of Human Tumours in Miçe. *Nature, Lond.*, **243**, 91.

HAMILTON, D. N. H. & CASTRO, J. E. (1972) Human Tumour Xenografts. *Br. J. Surg.*, **59**, 312.

JAMES, K. (1967) Anti-lymphocyte Antibody.—A Review. *Clin. & exp. Immunol.*, **2**, 615.

JEEJEEBHOY, H. F. (1967) Studies on the Mode of Action of Heterologous Anti-lymphocyte Sera. *Transplantation*, **5**, 273.

PHILLIPS, B. & GAZET, J-C. (1970) Transplantation of Primary Explants of Human Tumours to Mice Treated with Anti-lymphocyte Serum. *Br. J. Cancer*, **24**, 92.

POVLSEN, C. O. & RYGAARD, J. (1971) Heterotransplantation of Human Adenocarcinomas of the Colon and Rectum to the Mouse Mutant Nude. A Study of Nine Consecutive Transplantations. *Acta. path. microbiol. scand.*, **79**, 159.

TOOLAN, H. (1955) Subcutaneous Growth of Normal and Malignant Human Tissues in Heterologous Hosts. *Trans. N.Y. Acad. Sci.*, **17**, 589.